# Surgery for Mesothelioma After Radiation Therapy (SMART); A Single Institution Experience

**DOI:** 10.3389/fonc.2020.00392

**Published:** 2020-03-24

**Authors:** William G. Breen, Yolanda I. Garces, Kenneth R. Olivier, Sean S. Park, Kenneth W. Merrell, Francis C. Nichols, Tobias D. Peikert, Julian R. Molina, Aaron S. Mansfield, Anja C. Roden, Shanda H. Blackmon, Dennis A. Wigle

**Affiliations:** ^1^Department of Radiation Oncology, Mayo Clinic, Rochester, MN, United States; ^2^Division of Thoracic Surgery, Mayo Clinic, Rochester, MN, United States; ^3^Division of Pulmonary and Critical Care Medicine, Mayo Clinic, Rochester, MN, United States; ^4^Division of Medical Oncology, Mayo Clinic, Rochester, MN, United States; ^5^Department of Laboratory Medicine and Pathology, Mayo Clinic, Rochester, MN, United States

**Keywords:** mesothelioma, extrapleural pneumonectomy, thoracic oncology, hemithoracic radiation, neoadjuvant radiation

## Abstract

**Background:** The optimal treatment sequence for localized malignant pleural mesothelioma (MPM) is controversial. We aimed to assess outcomes and toxicities of treating localized MPM with neoadjuvant radiation therapy (RT) followed by extrapleural pneumonectomy (EPP).

**Methods:** Patients were enrolled on an institutional protocol of surgery for mesothelioma after radiation therapy (SMART) between June 2016 and May 2017. Eligible patients were adults with MPM localized to the ipsilateral pleura. Patients underwent staging with PET/CT, pleuroscopy, bronchoscopy/EBUS, mediastinoscopy, and laparoscopy. Five fractions of RT were delivered using intensity modulated radiation therapy (IMRT), with 30 Gy delivered to gross disease and 25 Gy to the entire pleura. EPP was performed 4–10 days following completion of RT.

**Results:** Five patients were treated on protocol. Median age was 62 years (range 36–66). Histology was epithelioid on initial biopsy in all patients, but one was found to have biphasic histology after surgery. Three patients had surgeon-assessed gross total resection, and two had gross residual disease. While all patients were clinically node negative by pretreatment staging, three had positive nodal disease at surgery. Patients were hospitalized for a median 24 days (range 5–69) following surgery. Two patients developed empyema, one of whom developed respiratory failure and subsequently renal failure requiring dialysis, while the other required multiple surgical debridements. Two patients developed atrial fibrillation with rapid ventricular response after surgery, one of whom developed acute respiratory distress requiring intubation and tracheostomy. At last follow-up, one patient died at 1.4 years after local and distant progression, two were alive with local and distant progression, and the remaining two were alive without evidence of disease at 0.1 and 2.7 years. Median time to progression was 9 months. Three patients received salvage chemotherapy.

**Conclusions:** SMART provided promising oncologic outcomes at the cost of significant treatment related morbidity. Due to the significant treatment associated morbidity and favorable treatment alternatives, we have not broadly adopted SMART at our institution.

## Introduction

Malignant pleural mesothelioma (MPM) is a rare but aggressive cancer of the pleural mesothelium. While incidence is decreasing in the United States due to decreased occupational asbestos exposure, 2,000–3,000 cases are still diagnosed per year, and prognosis remains poor (1, 2).

For patients with localized disease, guidelines support induction chemotherapy or initial surgical exploration, followed by surgical resection with or without adjuvant radiation (3). The optimal surgical approach is controversial, with many currently opting for pleurectomy/decortication (P/D) over EPP, due to concerns for excess morbidity with EPP (4). In a phase II trial, Rimner et al. report median survival approaching 2 years for patients treated with neoadjuvant chemotherapy, P/D, and adjuvant intensity-modulated radiation therapy (IMRT) (5).

While adjuvant radiation may improve outcomes after surgery, excess lung radiation can result in significant rates of toxicity, including fatal pneumonitis (6–9). An alternative approach of Surgery for Mesothelioma After Radiation Therapy (SMART) could result in decreased radiation-associated morbidity while providing favorable local control by immediately resecting the radiated lung (10). Pre-operative radiation also has the theoretical potential to sterilize microscopic disease and decrease local tumor seeding at surgery. Furthermore, because surgery is performed immediately after radiation and before the typical onset of significant radiation-related toxicity, this approach facilitates timely completion of both treatment modalities without treatment delays caused by toxicity. de Perrot and colleagues reported their institutional experience treating 62 patients with SMART, with a median survival of 3 years (11).

We initiated a protocol of SMART at our institution, along with thorough and modern staging procedures. Here we report our experience treating patients on this protocol.

## Materials and Methods

This study was approved by the institutional review board (IRB) and conducted in accordance with The Code of Ethics of the World Medical Association (Declaration of Helsinki). Informed consent was obtained from all patients.

Adults (age 18+) with localized epithelioid MPM who were deemed surgical candidates following a multidisciplinary evaluation were eligible to enroll on the protocol. All patients had baseline pulmonary function tests (PFT's).

Pre-therapy staging consisted of CT Chest, PET/CT, pleuroscopy, bronchoscopy with endobronchial ultrasound (EBUS), mediastinoscopy, and laparoscopy. Patients who had clinically localized, node negative disease following radiographic and surgical staging were allowed to enroll.

### Neoadjuvant Radiation

Radiation was delivered in five daily fractions over 1 week. The entire ipsilateral pleura was contoured as the clinical target volume (CTV) and expanded by 5 mm to define the planning target volume (PTV). The PTV was treated to 25 Gy. Gross tumor volume (GTV), as identified by PET-CT, was treated with a simultaneous integrated boost (SIB) to 30 Gy.

**Figure 1:**
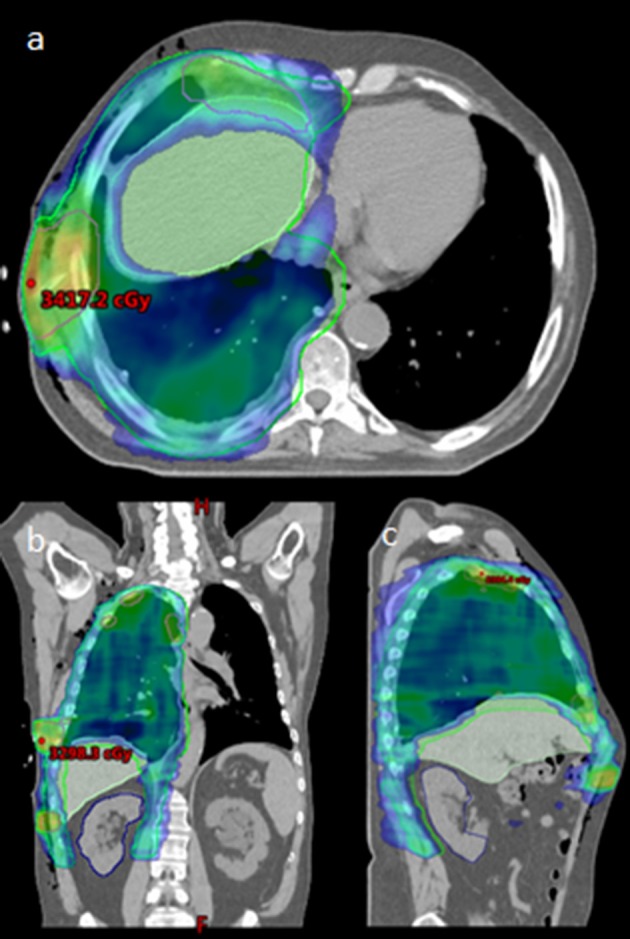
Radiation Plan for typical patient, **(a)**: axial, **(b)**: coronal, and **(c)**: sagittal. Maximum point dose indicated within boosted area of PET/CT identified gross disease.

Simulation was performed with 4D-CT (*n* = 2) or deep inspiration breath hold technique (*n* = 3) at the treating Radiation Oncologist's discretion. Patients were immobilized with arms up if possible. All patients were treated with IMRT, utilizing 1–2 isocenters, and 5–6 partial arcs. Cone-beam CT (CBCT) was used for daily image guidance. See Figure 1 for a typical radiation plan.

### Extrapleural Pneumonectomy

**Figure 2:**
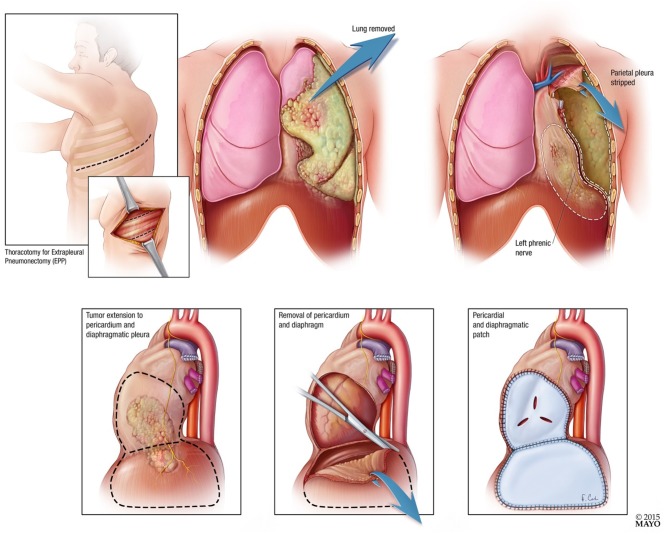
Diagrammatic representation of extrapleural pneumonectomy with pericardial and diaphragmatic resection and patch.

Patients underwent EPP 4–10 days after completing neoadjuvant RT. Surgery was performed using standard technique with resection and reconstruction of the pericardium and diaphragm (See Figure 2). Surgeons assessed resection status at the time of surgery. Extrapleural pneumonectomy included a muscle (latissimus) cutting thoracotomy, two intercostal incisions, harvesting a thymic fat pad or localized flap to cover the bronchial stump, pneumonectomy, resection, and reconstruction of the diaphragm, resection and reconstruction of the pericardium, and nodal dissection. The diaphragm was reconstructed with Gore-Tex Dualmesh. The pericardium was either reconstructed with Vicryl mesh or Gore-Tex mesh. Perforations were placed in the reconstructed pericardium so that tamponade did not occur postoperatively.

Histologic slides from all pneumonectomy specimens were re-reviewed by a thoracic pathologist (ACR) to confirm the diagnosis.

## Results

Five patients were enrolled on the protocol between June 2016 and May 2017. Three patients had left-sided disease, and 2 had right-sided disease. See Table 1 for patient characteristics.

**Table 1:** Patient characteristics.

**Patient**	**Age at Dx**	**Sex**	**ECOG PS**	**Asbestos exposure?**	**Smoking pack years**	**FEV1 (% expected)**	**DLCO (% Expected)**	**Histology on biopsy**	**cT stage**	**cN stage**
1	59	M	0	No	45	3.1 (87%)	25.5 (89%)	Epithelioid	3	0
2	62	M	0	Yes	0	2.47 (67%)	23.8 (83%)	Epithelioid	2	0
3	37	M	0	No	0	2.11 (60%)	20 (64%)	Epithelioid	2	0
4	66	M	0	Yes	5	2.72 (74%)	17.4 (61%)	Epithelioid	1	0
5	67	M	0	Yes	0	2.84 (82%)	18.9 (68%)	Epithelioid	1	0

*Staging per AJCC, 8th edition. Dx, diagnosis; ECOG PS, Eastern Cooperative Oncology Group Performance Status; FEV1, Forced expiratory volume over 1 s; DLCO, Diffusing capacity of lung for carbon monoxide*.

### Radiation

All patients successfully completed neoadjuvant RT. Target coverage was adequate for all patients. Median total lung V20Gy(%) was 42.5% (range 26.8–54.3). Median total lung mean dose and contralateral lung mean doses were 13.3 Gy (range 9.6–16.0) and 3.3 Gy (range 3.3–3.4), respectively. Median contralateral lung V7Gy(%) was 3.3% (range 0.1–5.3). Median heart mean dose was 14.6 Gy (12.5–15.2). Median esophagus mean dose was 14.6 Gy (13.7–18.1).

One patient developed grade 2 dermatitis after radiation. One patient had dyspnea and fatigue worsen from grade 1 to grade 2 from baseline to after radiation. Two patients developed grade 1 diarrhea. No other new or worsening toxicities were noted from baseline to post-treatment.

### Extrapleural Pneumonectomy

Pathologic T stage was pT3 (*n* = 4) or pT4 (*n* = 1). Pathologic N stage was pN0 (*n* = 2) or pN1 (*n* = 3). All three pN1 patients had only one positive lymph node, located in stations 4 (*n* = 1), 7 (*n* = 1), and 10 (*n* = 1). A median of 10 lymph nodes (9–39) were resected. Surgeon-assessed extent of resection was R1 in 3 patients and R2 in two patients. One patient had biphasic histology on surgical pathology (epithelioid component, 90%).

Median hospitalized days after surgery was 24 (range 5–69). All five patients experienced CTCAE grade 3+ toxicity. Four patients developed significant infections after surgery requiring antibiotics. Two patients developed empyema, one of whom developed respiratory failure and subsequently renal failure requiring dialysis, and required lifelong antibiotics. The other required multiple surgical debridements and long term antibiotics. Two patients developed atrial fibrillation with rapid ventricular response after surgery, one of whom developed acute respiratory distress requiring intubation and tracheostomy. One patient had significant post-operative blood loss requiring blood transfusions. There were no treatment related deaths within 90 days of surgery.

### Outcomes

See Table 2 for patient outcomes. One patient died of cardiopulmonary failure in the setting of progressive recurrent disease 1.4 years after initiation of treatment; the other four were alive at last follow-up. All three patients who had disease progression had simultaneous thoracic and extra-thoracic progression. One patient remains disease-free 2.7 years after treatment.

**Table 2:** Patient Outcomes.

**Patient**	**pT**	**pN**	**Extent of resection**	**Recurrence?**	**Site of initial recurrence**	**Time to recurrence (years)**	**Alive?**	**Overall survival (years)**
1	3	1	R1	No	N/A	N/A	Yes	0.3
2	3	0	R1	Local and Distant	Chest wall, mediastinal and axillary lymph nodes, peritoneal extension	2.5	Yes	2.6
3	3	0	R1	No	N/A	N/A	Yes	2.7
4	3	1	R2	Local and Distant	Chest wall, peri-splenic	0.5	No	1.4
5^*^	4	1	R2	Local and Distant	Chest wall, peritoneum	0.8	Yes	1.8

* *Patient had biphasic histology on surgical specimen*.

Initial salvage chemotherapy regimen was carboplatin and pemetrexed (*n* = 3). One patient went on to also receive salvage vinorelbine.

## Discussion

In this report, we describe five patients with MPM treated on a protocol of neoadjuvant high-dose, accelerated hemithoracic RT followed by EPP.

In our series, 60% of patients had nodal upstaging at surgery. This high rate of nodal upstaging occurred despite aggressive staging, with bronchoscopy and EBUS, mediastinoscopy, and PET/CT. These nodes were located at stations amenable to biopsy (4, 7, and 10) if they had been detected. Had these lymph nodes been detected during initial staging procedures, these patients would not have been considered for SMART. This is similar to the experience of de Perrot et al., who reported 52% of patients had positive mediastinal nodes at surgery after negative clinical staging (11). More accurate methods of staging are needed in order to better stratify patients for more aggressive treatment regimens.

Due to the small number of patients enrolled, no definitive conclusions regarding oncologic outcomes can be drawn. Still, only one patient has died to date, and one remains disease free more than 2.5 years after completing treatment. These early outcomes are encouraging.

Patients on this study experienced significant toxicity after EPP, which could have been exacerbated by decreased healing capacity due to neoadjuvant high-dose radiation. Compared to the 100% rate of grade 3+ post-operative toxicity observed on this study, previous reports of EPP for MPM without neoadjuvant radiation have reported 10–50% grade 3+ post-operative non- hematologic toxicity (4, 12).

In their experience with SMART, de Perrot and colleagues reported 24 of 62 patients (39%) experienced grade 3 complications, seven (11%) experienced grade 4 complications, and two (3%) experienced grade 5 complications. While our series is too small to meaningfully compare with their study, it is notable that they used multibeam IMRT, while we used partial arcs, potentially leading to different dosimetry in the contralateral lung and other non-target tissues. As a point of comparison, on the IMPRINT trial of IMRT following pleurectomy and decortication, Rimner et al. reported 11 grade 3 toxicities in 45 patients, with no grade 4–5 toxicities. The most common grade 3 toxicity was fatigue, reported in five patients (5).

Due to the significant morbidity associated with this protocol, and data indicating the safety and effectiveness of induction chemotherapy followed by P/D and adjuvant IMRT, we have hesitated to broadly adopt the SMART protocol at our institution, and instead routinely use P/D followed by adjuvant IMRT (5). Still, for certain patients, the SMART protocol could be a favorable option. Ideal candidates for SMART are patients with left-sided disease, excellent performance status and pulmonary function, and thorough staging with no lymph node or distant involvement (13).

Our study provides support to the feasibility of this innovative treatment regimen at highly specialized centers for properly selected patients, and also demonstrates the associated morbidity. There are several clear and significant limitations to our paper, primarily relating to the size of the cohort. One of the five patients was lost to follow-up shortly after discharge from the hospital, which is significant given the small cohort. This is not a randomized study, and therefore provides no insights into the comparative effectiveness of SMART vs. P/D followed by adjuvant IMRT.

Prospective studies are needed to better define the efficacy and safety of SMART. The SMARTER clinical trial (NCT04028570) aims to find the maximum safely tolerated neoadjuvant radiation dose to the hemithorax, with a boost to gross disease. This trial is currently accruing patients, and will provide important prospective safety evidence for this approach. Newer radiation modalities, including proton beam therapy, could potentially improve the therapeutic ratio by decreasing radiation dose to the contralateral lung, heart, and other non-target tissues. Integration of systemic therapies, such as immune checkpoint inhibitors, may also play a role in treatment paradigms in the future.

SMART may be a feasible treatment option for some patients, potentially providing good oncologic outcomes at the cost of significant morbidity.

## Data Availability Statement

Datasets are available on request. The raw data supporting the conclusions of this article will be made available by the authors, without undue reservation, to any qualified researcher.

## Ethics Statement

The studies involving human participants were reviewed and approved by Mayo Clinic Institutional Review Board (IRB). The patients/participants provided their written informed consent to participate in this study. Written informed consent was obtained from the individual(s) for the publication of any potentially identifiable images or data included in this article.

## Author Contributions

DW and SB developed the protocol and directed treatment. AR reviewed all pathologic specimens to confirm histology for this report. WB collected and analyzed data, and prepared the manuscript. All authors reviewed and approved of the manuscript, and were involved in patient care.

### Conflict of Interest

The authors declare that the research was conducted in the absence of any commercial or financial relationships that could be construed as a potential conflict of interest.
